# Small-Molecule Induction Promotes Corneal Endothelial Cell Differentiation From Human iPS Cells

**DOI:** 10.3389/fbioe.2021.788987

**Published:** 2021-12-15

**Authors:** Jie Chen, Qingjian Ou, Zhe Wang, Yifan Liu, Shuqin Hu, Yumeilan Liu, Haibin Tian, Jingying Xu, Furong Gao, Lixia Lu, Caixia Jin, Guo-Tong Xu, Hong-Ping Cui

**Affiliations:** ^1^ Department of Ophthalmology, Shanghai East Hospital, Tongji University School of Medicine, Shanghai, China; ^2^ Department of Ophthalmology, Shanghai Tenth People’s Hospital, Tongji University School of Medicine, Shanghai, China; ^3^ Department of Biochemistry and Molecular Biology, School of Medicine, Tongji University, Shanghai, China

**Keywords:** corneal endothelial cell, small molecule screening, human induced pluripotent stem cell (hiPSC), neural crest cell, AT13148, A769662

## Abstract

**Purpose:** Corneal endothelial cells (CECs) serve as a barrier and foothold for the corneal stroma to maintain the function and transparency of the cornea. Loss of CECs during aging or disease states leads to blindness, and cell replacement therapy using either donated or artificially differentiated CECs remains the only curative approach.

**Methods:** Human induced pluripotent stem cells (hiPSCs) that were cultured in chemically defined medium were induced with dual-SMAD inhibition to differentiate into neural crest cells (NCCs). A small-molecule library was screened to differentiate the NCCs into corneal endothelial-like cells. The characteristics of these cells were identified with real-time PCR and immunofluorescence. Western blotting was applied to detect the signaling pathways and key factors regulated by the small molecules.

**Results:** We developed an effective protocol to differentiate hiPSCs into CECs with defined small molecules. The hiPSC-CECs were characterized by ZO-1, AQP1, Vimentin and Na^+^/K^+^-ATPase. Based on our small-molecule screen, we identified a small-molecule combination, A769662 and AT13148, that enabled the most efficient production of CECs. The combination of A769662 and AT13148 upregulated the PKA/AKT signaling pathway, FOXO1 and PITX2 to promote the conversion of NCCs to CECs.

**Conclusion:** We established an efficient small molecule-based method to differentiate hiPSCs into corneal endothelial-like cells, which might facilitate drug discovery and the development of cell-based therapies for corneal diseases.

## Introduction

Corneal endothelial cells (CECs) form the innermost cellular monolayer of the cornea and serve as a barrier and foothold for the corneal stroma to maintain the function and transparency of the cornea (Bahn et al., 1984; Bonanno, 2012). CECs are gradually lost with age and cannot proliferate *in vivo* in response to disease states such as corneal endothelial dystrophies and surgical trauma (Bourne, 2003; Ong Tone et al., 2021). If the cell density drops below a critical level, the pump and barrier functions of CECs fail, which results in corneal decompensation and ultimately in loss of vision (Joyce, 2003). Regarding the nonproliferative properties of CECs, there are no treatments to cure diseases with CEC dysfunction except for corneal transplantation. Descemet’s membrane endothelial keratoplasty (DMEK) with transplantation of the corneal endothelium and Descemet’s membrane can recover visual acuity (Gain et al., 2016; Schlögl et al., 2016; Wacker et al., 2016). The lack of human corneal donors has limited the development and application of various corneal transplant surgeries. More alternative corneal products and sources are urgently needed for clinical application. It is encouraging that injection of cultured CECs and a rho-associated protein kinase (ROCK) inhibitor into patients’ anterior chambers was found to increase CEC density in 11 patients with bullous keratopathy after 24 weeks (Kinoshita et al., 2018). Transplantation of pluripotent stem cell derived CECs to the anterior chamber of rabbits or monkeys can rescue the corneal edema caused with dysfunction of corneal endothelial cells (Ali et al., 2021; Hatou et al., 2021).

The corneal endothelium and stroma are derived from neural crest cells (NCCs) during embryonic development (Walker et al., 2020). In recent years, several groups have generated CECs from human pluripotent stem cells or human induced pluripotent stem cells (hiPSCs) through NCCs (Chambers et al., 2009; Chen et al., 2015; Zhao and Afshari, 2016). For example, an approach involving treatment of human embryonic stem cells (hESCs) with different conditioned media to induce directed endothelial differentiation has been introduced (McCabe et al., 2015). Additionally, a method of small molecule-based conversion of mouse embryonic fibroblasts into NCCs and functional CECs has been developed, which adds importance to the strategy of small molecule-mediated induction for CEC generation (Pan et al., 2021).

Although multiple methods of CEC differentiation have been developed, low efficiency and long protocol durations remain challenges. Here, we report an efficient small molecule-based method to differentiate hiPSCs into CECs. We screened a new cocktail of small molecules designated stem cell differentiation molecules and identified a small-molecule combination, A769662 and AT13148, that enabled the most efficient production of CECs. The CECs showed gene expression profiles similar to those of human CECs. Our findings provide a new approach for the generation of functional corneal endothelial-like cells and may facilitate drug discovery and the development of cell-based therapies for corneal diseases.

## Materials and Methods

### Cell Culture

This research was approved by the ethics committee of East Hospital Affiliated with Tongji University in Shanghai. The human iPS cell lines (hiPSCs) used for *in vitro* differentiation was a gift from Professor Jin Ying (Chinese Academy of Sciences, China). The hiPSCs were cultured in basal medium [BM; DMEM/F12 medium (Thermofisher, United States)] supplemented with 1 × N2(Thermofisher, United States), 1 ×  B27(Thermofisher, United States), 0.1 mM minimum essential medium with nonessential amino acids (MEM NEAA; Gibco, United States), and 0.1 mM 2-mercaptoethanol (Gibco, United States) containing 10 ng/ml BFGF (R&D Systems, United States), as previously described (Jin et al., 2018). For the human corneal endothelial cells (HCEC-B4G12) (Zeye Culture Collection, China) cultured in DMEM/F12 medium (Thermofisher) supplemented with 10% fetal bovine serum (FBS, ExCell Bio, China). The cultures were incubated in a humidified atmosphere of 5% CO_2_ at 37°C. The medium was changed every day.

### Differentiation of hiPSCs Into CECs

**FIGURE 1:**
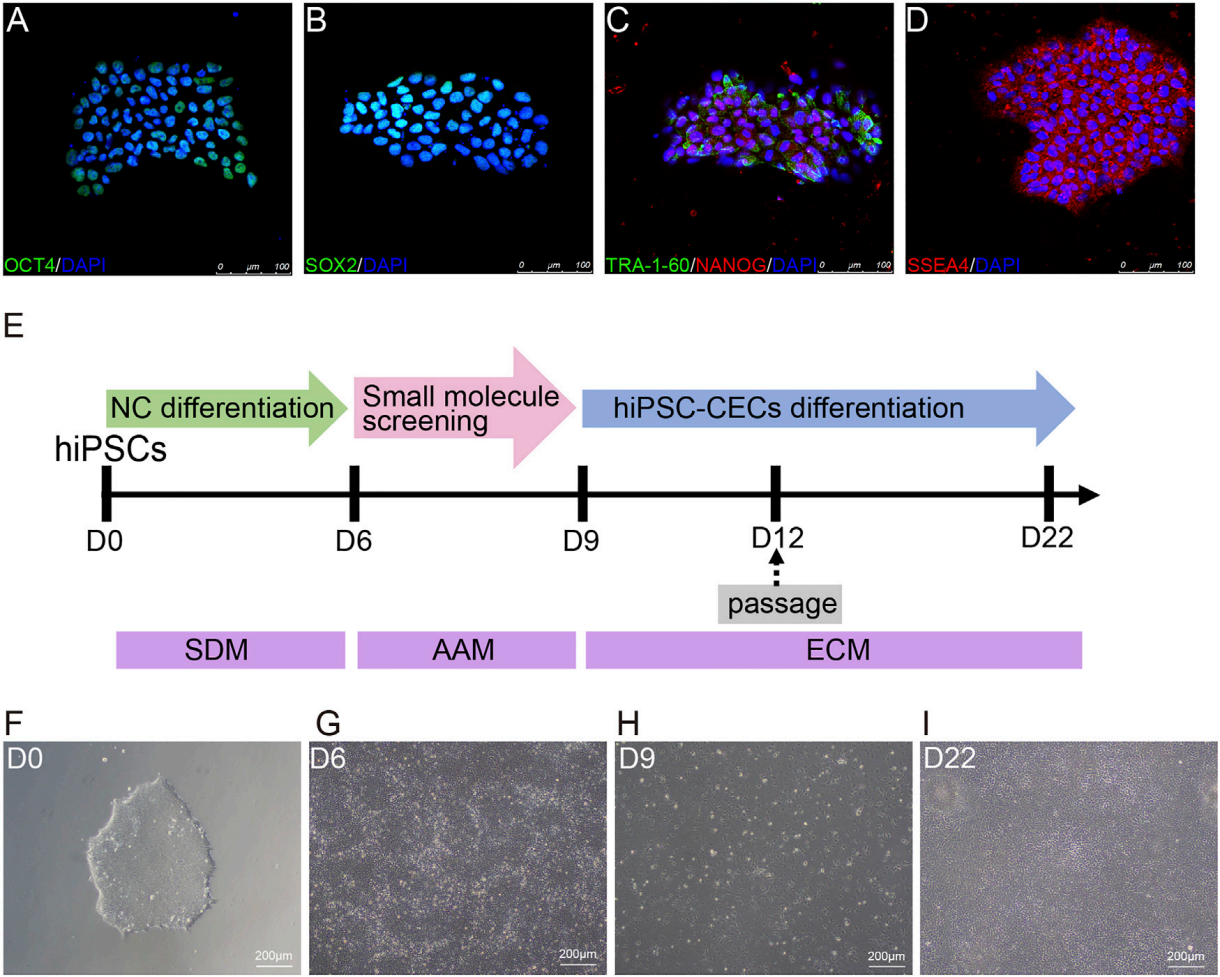
Differentiation of hiPSCs into human CECs with CDM. **(A–D)** Immunostaining of hiPSC clones positive for OCT4, SOX2, TRA-160, NANOG, and SSEA4. **(E)** Schematic diagram of the generation of CECs. Small molecules were sequentially used to induce hiPSCs to differentiate into CECs. SB431542 and DMH1 were used for 6 days; A769662 and AT13148 were selected from 30 small molecules and used for 3 days; and EGF and CHIR-99021 were used for 13 days. **(F)** Morphological features of the hiPSC clones on plates coated with Matrigel under a light microscope. **(G–I)** Brightfield images of cells at different stages during CEC cell induction.

There were three stages of CEC differentiation (Figure 1E). In the first stage, the hiPSCs were cultured with 2 µM SB431542 (Selleck, United States) and 2 µM DMH1 (Selleck, United States) for 6 days with daily medium changes to differentiate into NCCs. In the second stage, 30 small molecules related to stem cell differentiation and corneal development were screened on hiPSC-derived NCCs. On day 6, the cells were grown in chemically defined medium (CDM) supplemented with small-molecule compounds for 3 days during the second stage. In the last stage, 20 ng/ml epidermal growth factor (EGF, Thermofisher, United States) and 2 µM CHIR99021 (Selleck, United States) were added to the medium, and the cells were cultured for an additional 13 days with daily medium changes.

### Screening of Small Molecules

Firstly, the hiPSC-NCCs were passaged to the 24-well cell culture plate and cultured with the small molecules respectively as shown in Table 1. The total RNA was extracted to detect the expression of relative genes. The significant high expression of the relative genes was used to pick up the candidate molecule. Subsequently, the combination of two types of candidate molecules was applied to treat the hiPSC-NCCs. The expression level of the relative genes was applied to screen and evaluate the combination of the molecules.

**TABLE 1:** Chemical library involved in this study.

ID	Name	Target	Pathway	Final Conc
1	Fenofibrate	MMP inhibitor; PPAR agonist	Metabolism; proteases/proteasome	2 μM
2	Ganetespib (STA-9090)	HSP inhibitor	Cell cycle/checkpoint	0.5 μM
3	WY-14643	PPAR activator	Metabolism	2 μM
4	Tenovin-6	p53 activator; sirtuin inhibitor	Apoptosis	5 μM
5	SRT1720	Sirtuin inhibitor	Chromatin/epigenetic	2.5 μM
6	A 769662	AMPK activator	PI3K/Akt/mTOR signaling	10 μM
7	UNC2881	TAM receptor inhibitor	Tyrosine kinase/adaptors	1 μM
8	AL082D06	Glucocorticoid receptor antagonist	Endocrinology/hormones	2 μM
9	(+)-Matrine	Opioid receptor agonist	Neuroscience	2 μM
10	Wiskostatin	N-WASP inhibitor	Cell cycle/checkpoint	1 μM
11	Purmorphamine	Hedgehog/smoothened receptor antagonist	Stem cell	1 μM
12	GTPL5846	GPR agonist	GPCR/G protein	2 μM
13	Kartogenin	TGF-beta/Smad activator	Immunology/inflammation	1 μM
14	APD 668	GPR inhibitor	GPCR/G protein	2 μM
15	Forsythin	p38 MAPK inhibitor	MAPK signaling	2 μM
16	AT13148	Akt inhibitor; ROCK inhibitor; S6 kinase inhibitor; PKA inhibitor	PI3K/Akt/mTOR signaling	10 μM
17	IC-87114	PI3K inhibitor	PI3K/Akt/mTOR signaling	2 μM
18	BPR1J-097	FLT3 inhibitor	Tyrosine kinase/adaptors	1 μM
19	1NM-PP1	Src inhibitor	Tyrosine kinase/adaptors	2 μM
20	Hydroxyprogesterone	Estrogen/Progestogen Receptor agonist	Endocrinology/hormones	2 μM
21	Bucladesine	MAPK activator	MAPK signaling	2 μM
22	7,8-Dihydroxyflavone	Trk receptor inhibitor	Tyrosine kinase/adaptors	2 μM
23	Fisetin	Sirtuin activator	Chromatin/epigenetic	10 μM
24	Antrapurol	AMPK activator	PI3K/Akt/mTOR signaling	10 μM
25	Panobinostat (LBH589)	HDAC inhibitor	Chromatin/epigenetic	50 nM
26	LY-2874455	FGFR inhibitor; VEGFR inhibitor	Tyrosine kinase/adaptors	1 μM
27	Ginsenoside Rg2	GSK-3 inhibitor	PI3K/Akt/mTOR signaling	5 μM
28	Crenolanib	PDGFR inhibitor	Tyrosine kinase/adaptors	1 μM
29	Vismodegib (GDC-0449)	Hedgehog/smoothened receptor antagonist	Stem cell	20 μM
30	Flavopiridol (Alvocidib) hydrochloride	CDK inhibitor	Cell cycle/checkpoint	50 nM

### Cell Counting Kit-8 (CCK-8) Assay

The cultured neural crest cells (NCCs) were passaged to the 96-well plated for 24 h. The NCCs were treated with chemical molecules of different concentrations for another 24 h. The cell viability was detected with cell counting kit-8 reagent (TargetMol, United States) according to the manufacturer’s instructions.

### Quantitative Real-Time PCR (qRT-PCR)

RNA was extracted from cells with RNAiso Plus reagent (Cat. No. 9019, Takara, Japan) and chloroform. The concentration of RNA was measured with a NanoDrop spectrophotometer. Approximately 1.0 µg of total RNA was reverse-transcribed into complementary DNA with PrimeScript RT Master Mix (RR036A, Takara, Japan). qRT-PCR was run with SYBR Green PCR Master Mix (Tiangen Biotech, China) and the following cycling parameters: denaturation at 95°C for 5 min followed by 39 cycles of 95°C for 30 s and 60°C for 30 s. The relative expression level of each gene was analyzed using the 2^−ΔΔCT^ method. The primers used in this study are listed in Supplementary Table S1.

### Immunofluorescence Staining

The cells were fixed with 4% paraformaldehyde (Sigma-Aldrich, Germany), permeabilized with 0.1% Triton X-100 (Sigma-Aldrich) in PBS for 10 min, washed 3 times for 5 min/wash with PBS, and then blocked with 3% BSA (Sangon Biotech, China) in PBS (Sangon Biotech, China) for 1 h at room temperature. The cells were incubated with the primary antibodies (Supplementary Table S2) and then incubated overnight at 4°C. Then, they were washed 3 times for 5 min/wash with PBS and incubated with the following fluorescent secondary antibodies for 1 h at room temperature: Alexa Fluor 555-conjugated donkey anti-mouse (1:500, Thermofisher, United States) and Alexa Fluor 488-conjugated donkey anti-mouse (1:500, ThermoFisher, United States). After three washes for at least 10 min each, the cells were exposed to DAPI (Sigma-Aldrich, Germany) for 5 min at room temperature to visualize nuclei. The samples were washed 3 times for 5 min each, and then pictures were taken with a Olympus microscope (Olympus, Japan).

### Western Blot Analysis

Total proteins of cells were extracted using RIPA lysis buffer (Beyotime, China) supplemented with protease and phosphatase inhibitor cocktails (TargetMol, United States) on ice, and the protein concentrations were then determined with a BCA assay (Pierce, United States). Twenty micrograms of protein was run on a 10–15% polyacrylamide gel and transferred to a polyvinylidene fluoride (PVDF) membrane (Millipore, Germany). The blots were blocked with 5% BSA in TBS +0.1% tween 20 and incubated with primary antibodies (Supplementary Table S2) in 5% BSA overnight at 4°C. Then, the cells were incubated with the corresponding HRP-conjugated secondary antibodies (Proteintech, United States) for 1 h at room temperature. Images of the blots were obtained by using a Tanon system with enhanced chemiluminescence (ECL) reagent (Thermofisher, United States).

### Statistical Analysis

All data are expressed as the mean ± SEM. All analyses were performed with GraphPad Prism 9.0 software. One-way ANOVA was employed for the statistical comparisons. A value of *p* < 0.05 was considered to indicate statistical significance.

## Results

### Scheme of Differentiation of hiPSCs Into CECs

hiPSCs were cultured in CDM on Matrigel-coated cell culture plates and showed the typical cell morphology of pluripotent stem cells (Figure 1F). Moreover, the cultured hiPSCs were confirmed to express pluripotency markers such as OCT4, SOX2, NANOG, SSEA4 and TRA-1-60 by immunofluorescence (Figures 1A–D).

In this study, we induced hiPSCs to differentiate into neural crest cells (NCCs) and CECs in an orderly manner with CDM, as shown in Figure 1E. Briefly, hiPSCs were induced via dual-SMAD inhibition with SB431542 (to inhibit TGF-β-Smad-2/3 signaling) and DMH1 (to inhibit BMP-Smad1/5/8 signaling) to differentiate into NCCs (Figure 1G) for 6 days. Then, the NCCs were screened with small molecules for another 3 days to induce them to differentiate into cornea-destined cells and continuously cultured to differentiate them into CECs for another 13 days (Figures 1H,I).

### Differentiation of hiPSCs Into NCCs

**FIGURE 2:**
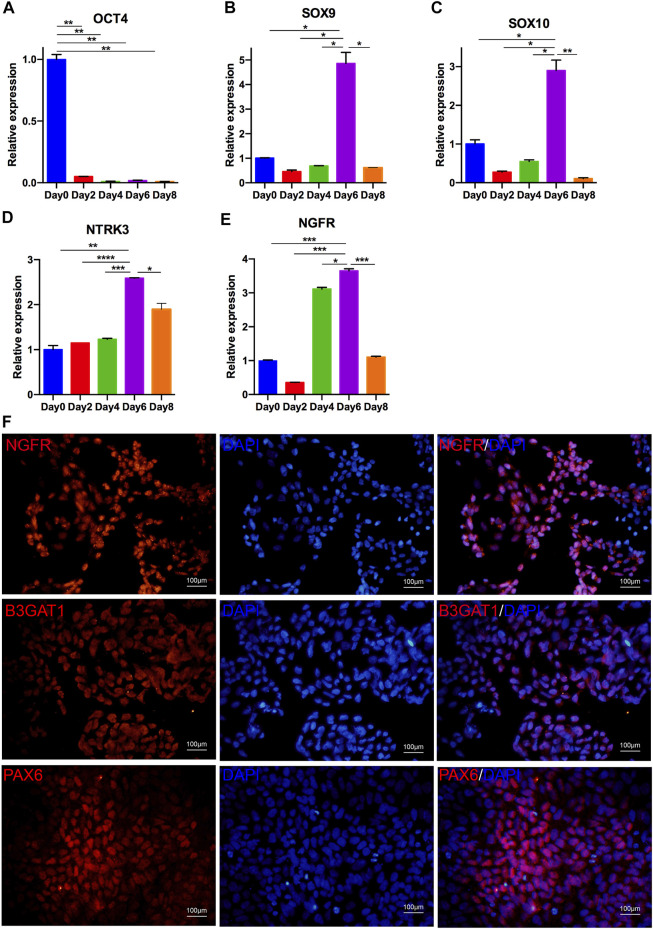
Differentiation of hiPSCs into NCCs. **(A–E)** Optimal time for NCC culture. qRT-PCR analysis indicated that the expression of OCT4 was downregulated after 2 days of differentiation; the expression of NCC markers, including SOX9, SOX10, NTRK3 and NGFR, was upregulated in differentiated NCCs. The expression of these genes began to increase at day 6 and decrease at day 8. **(F)** Immunostaining of NCCs positive for NGFR, B3GAT1, and PAX6.

NCCs, also named neural progenitor cells, are the original source of cornea-destined cells. Thus, we initially adopted a procedure to induce hiPSCs to differentiate into NCCs with dual-SMAD inhibition *via* SB431542 and DMH1. Initially, we detected the transcription levels at four time points to confirm the best time course for neural conversion of hiPSCs in the CDM. As shown in Figure 2A, the pluripotency marker OCT4 was significantly downregulated on day 2. Simultaneously, NCC-related markers (SOX9, SOX10, NTRK3, and NGFR) were upregulated on day 6 (Figures 2B–E).

The induced cells on day 6 showed a loss of hiPSC colonization and neuronal epithelial morphology (Figure 1G). We further analyzed the expression of the NCC-related genes PAX6, NGFR and B3GAT1 (Cheung et al., 2014). As shown in Figure 2F, almost all of the cells differentiated from the hiPSCs expressed NGFR, B3GAT1 and PAX6 on day 6. Thus, 6 days was selected for the induction of the NCC differentiation.

### Screening of Small Molecules to Promote the Conversion of NCCs Into CECs

**FIGURE 3:**
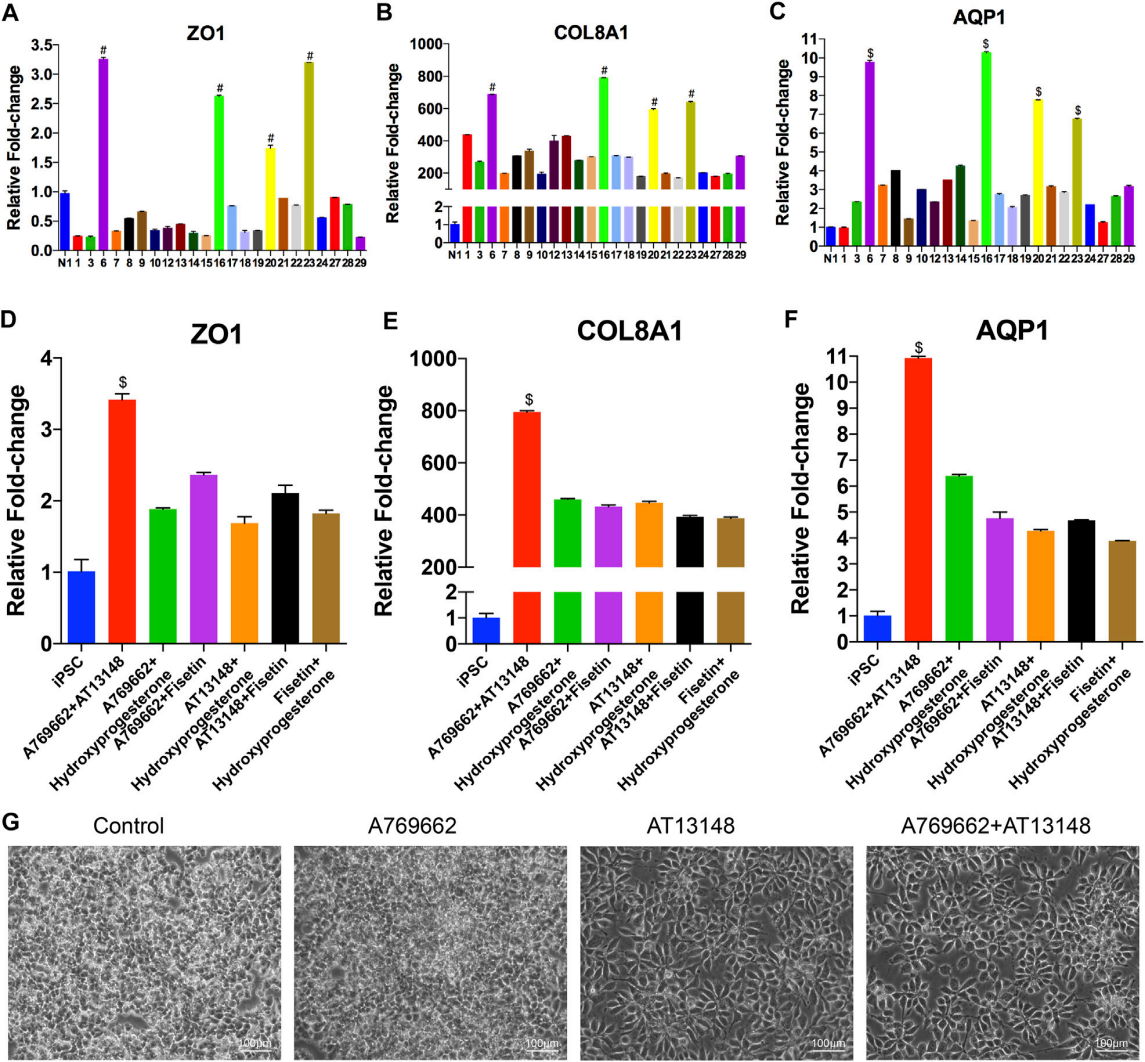
Screening of small molecules for the induction of CEC differentiation from NCCs. **(A–F)** mRNA expression of CEC markers after induction with different small molecules. At day 9 of CEC cell differentiation, 30 compounds related to the cornea were chosen from a stem cell differentiation compound library and screened. **(G)** Brightfield images of cells after induction with different small molecules, including CDM, A769662, AT13148, A769662 and AT13148.

To identify small molecules facilitating the induction of NCCs to differentiate into CEC destined cells, we built a chemical library of 30 small molecules that targeted almost all the key pathways of stem cell differentiation and corneal development (Table 1). The final concentration of each compound added to the medium was based on previously reported data and testing for the ED50s in the NCCs *via* Cell Counting Kit-8 (CCK-8) assay (Miner et al., 2003; Bouzakri et al., 2004; Moreno et al., 2008; Johnson et al., 2012; Yap et al., 2012; Cho et al., 2013; Han et al., 2014; Schelleman et al., 2014; Kampa-Schittenhelm et al., 2017; Pinkosky et al., 2020; Zhang et al., 2020; Gampala et al., 2021; Ma et al., 2021). For initial screening, NCCs derived from dual-SMAD inhibition of hiPSCs for 6 days were cultured in a 24-well plate and treated with small molecules for another 3 days. 7 compounds, numbered 2 [ganetespib (STA-9090)], 4 (tenovin-6), 5 (SRT1720), 11 (purmorphamine), 25 [panobinostat (LBH589)], 26 (LY-2874455), and 30 (flavopiridol hydrochloride), were excluded, as almost of the cells died by the final time point. The effective candidate compounds were selected according to the transcriptome levels of CEC markers (AQP1, ZO-1, and COL8A1) at the final time point, as determined by qRT-PCR. Then, compounds 6 (A769662), 16 (AT13148), 20 (hydroxyprogesterone) and 23 (fisetin) were selected as compounds inducing high expression of AQP1, ZO1 and COL8A1 (Figures 3A–C). We further confirmed whether the combination of two of the four candidates improved the expression of CEC markers. The combination of 6 (A769662) and 16 (AT13148) clearly promoted the expression of AQP1, ZO1 and COL8A1 (Figures 3D–F). Additionally, the cell morphology after treatment with the combination of A769662 and AT13148 was more homogeneous than that after treatment with A769662 or AT13148 separately (Figure 3G). Taken together, these data demonstrate that the combination of AT13148 and A769662 promotes corneal endothelial differentiation from NCCs.

### Characterization of CECs Differentiated From hiPSCs by the Small Molecule-Based Method

**FIGURE 4:**
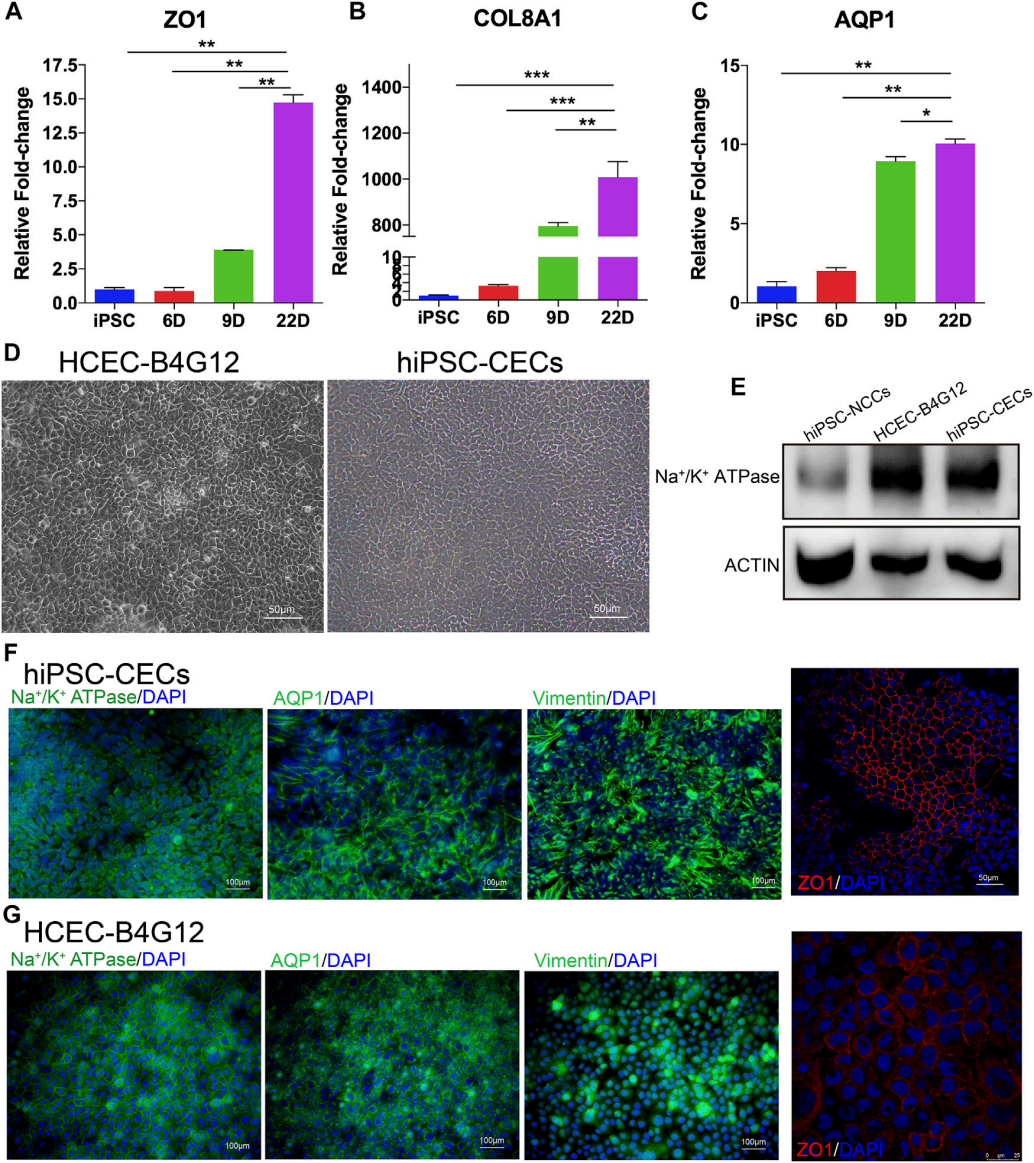
Characterization of CECs derived from hiPSCs. **(A–C)** mRNA expression of CEC markers, including ZO1, COL8A1 and AQP1 during CEC cell induction. **(D)** Brightfield images of human CEC cell lines (B4G12) and hiPSC-CECs. **(E)** Western blot results indicating that the expression of Na^+^/K^+^-ATPase was higher than that in NCCs (the control). **(F,G)** Immunofluorescence staining showing that hiPSC-derived CECs **(F)** and human CEC cell lines **(G)** express Na^+^/K^+^-ATPase, AQP1, ZO-1 and vimentin.

We further identified the hiPSC derived CECs (hiPSC-CECs) with the qRT-PCR, western blot and immunofluorescence. hiPSC-CECs showed a tightly packed hexagonal/polygonal appearance and similar to CEC morphology on day 22 (Figure 4D). Compared with hiPSCs and CECs at previous stages (day 6 and day 9), hiPSC-derived CECs highly expressed ZO1, AQP1 and COL8A1, as determined by qRT-PCR (Figures 4A–C). Additionally, the expression of Na^+^/K^+^-ATPase was significantly expressed in the hiPSC-CECs compared to the hiPSC-NCCs (Figure 4E). The hiPSC-CECs expressed Na^+^/K^+^-ATPase, AQP1, ZO1 and vimentin. The cell connection protein ZO1 was regularly distributed on the edges of the cells (Figure 4F). The protein expression and distribution is similar with the human CEC cell lines (B4G12) (Figure 4G). Thus, we considered the protocol shown the successful differentiation of hiPSC-CECs from hiPSC.

### Signaling Pathway Regulation by AT13148 and A769662

The combination of AT13148 and A769662 clearly promoted the differentiation of NCCs into CECs. To further elucidate the mechanism of AT13148 and A769662 in the differentiation of CECs from hiPSCs. We analyzed the protein level of the NCCs treated with AT13148 and A769662. A769662, similar to activators of adenosine monophosphate (AMP), is a selective and effective molecule that activates AMP kinase (AMPK), maintains energy balance, preserves endothelial cell vitality, and enhances endothelial cell differentiation and migration (Göransson et al., 2007). AT13148 is an ATP-competitive inhibitor of multiple AGC kinases, including AKT, phosphoinositide-dependent kinase 1 (PDK1), p70S6 kinase (p70S6K), p90 ribosomal S6 kinase (RSK), glycogen synthase kinase 3β (GSK-3β) and ROCK.

**FIGURE 5:**
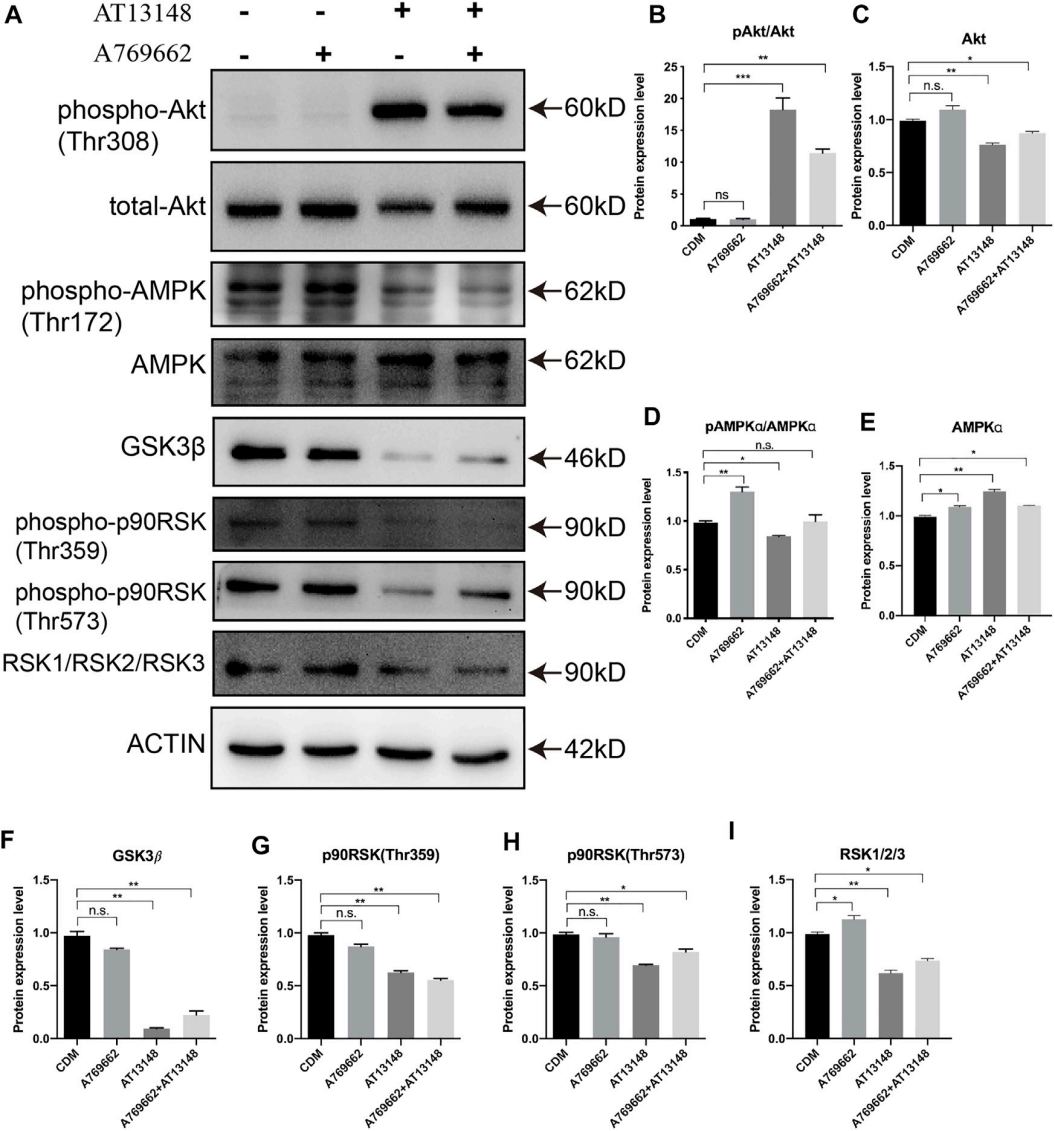
Western blot analysis of the key signaling pathways upon A769662 and AT13148 treatment. **(A)** Lanes for AT13148-and A769662-related signaling pathways. **(B–I)** Bar graph showing the relative protein expression levels from the western blot.

We detected the related key signaling proteins to explore the potential mechanism, as shown in Figure 5A. The phosphorylation level of Akt was significantly upregulated in the AT13148-treated group and the combination-treated group, while the control group and the A769662-treated group had almost no phosphorylation of AKT. The total Akt levels in the AT13148 and combination groups were downregulated (Figures 5B,C). It has previously been reported that AKT inhibitors can lead to hyperphosphorylation of AKT on regulatory sites (including Thr308), leading to AKT activation that can counteract the effects of small-molecule inhibitors (Okuzumi et al., 2009). A769662 can significantly increase the phosphorylation level of the Thr172 site of AMPK. However, AT13148 inhibited the phosphorylation of AMPK and upregulated total AMPK (Figures 5D,E). Additionally, the expression of GSK3β, the phosphorylation of p90RSK (Thr359 and Thr573) and the expression of total RSK were inhibited by AT13148. This inhibition by AT13148 was partially counteracted by A769662 (Figures 5F–I).

### Dynamic Regulation of Key Transcription Factors With AT13148 and A769662

The above results suggest that AT13148 and A769662 can upregulate AMPK and PI3K/AKT signaling pathways during the differentiation of NCCs into CECs. To explore the regulation of the key transcription factors in this process, FOXO, PAX6, PITX2 and FOXC1 were detected by western blotting. The forkhead box class O (FOXO) family, which includes FOXO1, FOXO3a, FOXO4, and FOXO6, can be regulated by the phosphoinositol-3-kinase (PI3K)-Akt signaling pathway and specifically activate a coordinated transcriptional program to regulate developmental processes and energy metabolism in embryo development (Hosaka et al., 2004; Maiese, 2015; Martins et al., 2016).

**FIGURE 6:**
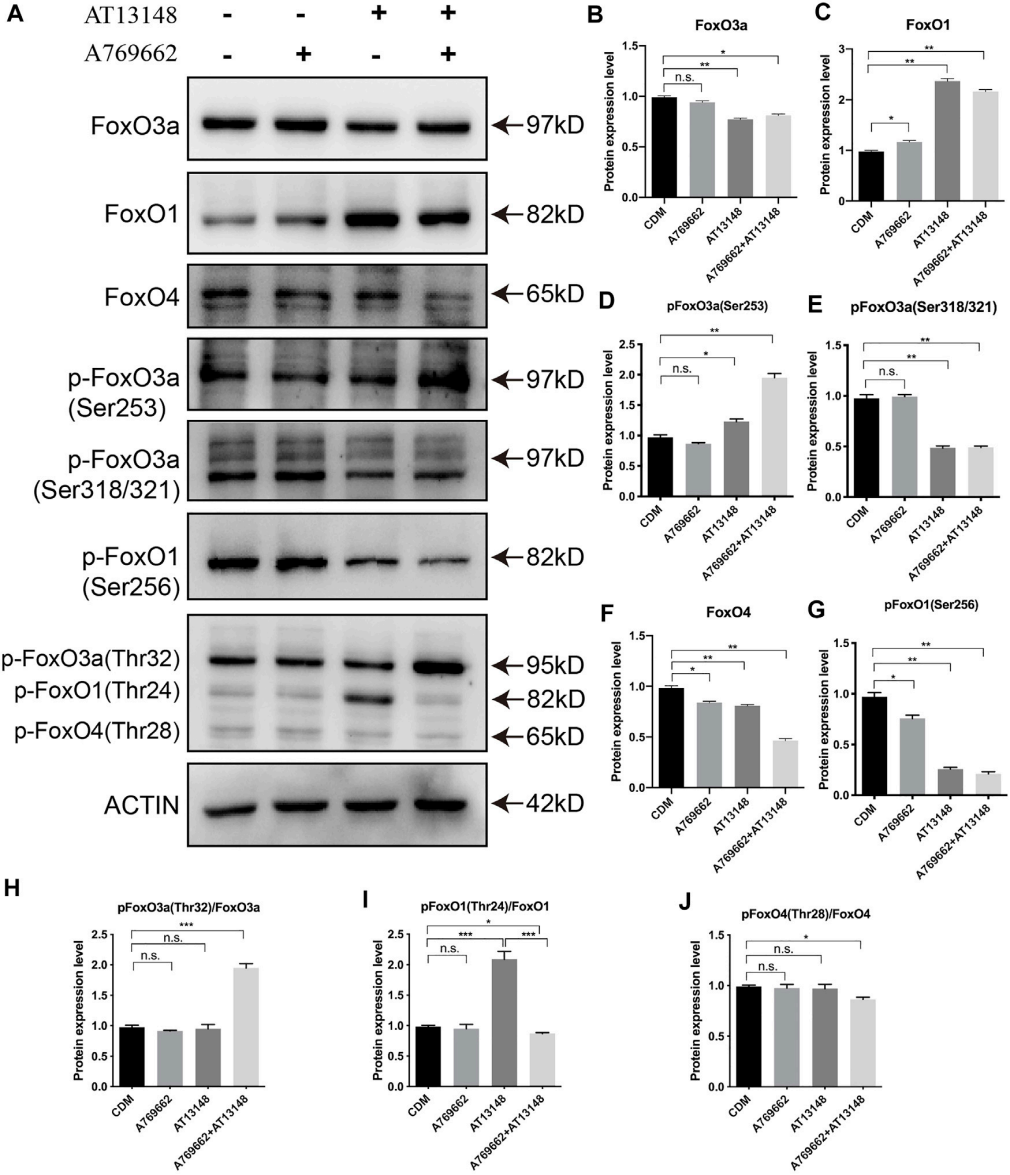
Dynamic regulation of the FOXO family with A769662 and AT13148. **(A)** Expression and phosphorylation of FOXO family members. **(B–J)** Bar graph showing the relative protein expression levels from the western blot.

As shown in Figure 6, AT13148 inhibited the expression of FoxO3a and FoxO4 and promoted the expression of FoxO1. The combination of AT13148 and A769662 downregulated the expression of FoxO3a and FoxO4 and upregulated the expression of FoxO1. With regard to the phosphorylation levels of FoxO1, FoxO3a and FoxO4, the phosphorylation at sites Ser253 and Thr32 of FoxO3a was significantly upregulated compared with that in the control group. Moreover, compared to AT13148, phosphorylation at the Ser256 and Thr24 sites of FoxO1 and the Ser318 and Ser321 sites of FoxO3a was inhibited by the combination of AT13148 and A769662. Phosphorylation at the Thr28 site of FoxO4 was inhibited by the combination treatment. Thus, the combination of AT13148 and A769662 can upregulate FoxO1 by inhibiting the phosphorylation of FoxO1 at Ser256 and Thr24. In addition, the combination of AT13148 and A769662 can inhibit FoxO3a and FoxO4 expression by upregulating the phosphorylation of FoxO3a at Ser253 and Thr32 and FoxO4 at Thr28.

**FIGURE 7:**
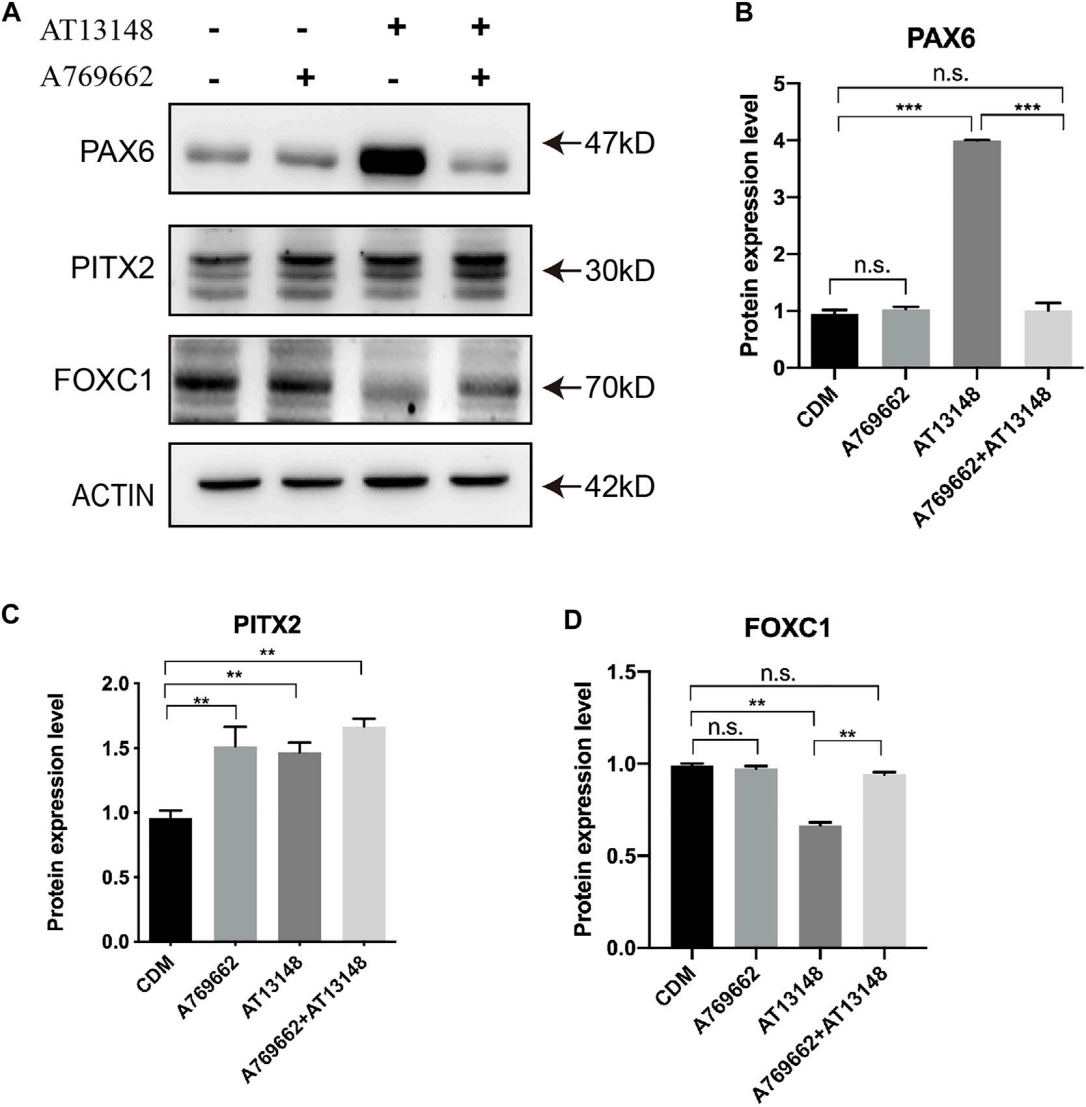
Dynamic regulation of key transcription factors with A769662 and AT13148. **(A)** Expression of key transcription factors. **(B–D)** Bar graph showing the relative protein expression levels from the western blot.

The key transcription factors consist of PAX6, PITX2 and FOXC1, which are required for the development of the ocular anterior segment and corneal endothelium (Zavala et al., 2013). As shown in Figure 7A, PITX2 was upregulated by AT13148, A769662 or the combination treatment. With regard to the expression of FOXC1, AT13148 partially inhibited the expression of FOXC1, while the combination of AT13148 with A769662 recovered the expression of FOXC1(Figures 7B–D). The combination of AT13148 and A769662 did not change the level of PAX6, while AT13148 significantly upregulated the expression of PAX6.

## Discussion

hiPSCs can be generated from multiple types of somatic cells and are a promising source for CEC differentiation in regenerative medicine. Compared to hESCs, hiPSCs have the advantage of avoiding ethical problems. Both dermal fibroblasts and peripheral blood mononuclear cell sourced hiPSC could be differentiated into CECs which proteome character was similar with the human cornea endothelium tissue *in vitro* (Ali et al., 2018; Wagoner et al., 2018). We expect to develop a chemical defined medium (CDM) protocol to differentiate hiPSCs into CECs. A chemically defined protocol would be more realistic and repeatable to obtain abundant CECs for clinical applications.

Generally, CECs and corneal stromal cells are derived from NCCs during the development of the ocular anterior segment. It has previously been reported that NCCs are derived from pluripotent stem cells via dual-SMAD inhibition, including inhibition of both TGFβ-Smad 2/3 signaling and BMP-Smad 1/5/8 signaling (Chambers et al., 2009). In this study, we also induced hiPSCs to differentiate into NCCs *via* dual-SMAD inhibition (McCabe et al., 2015). Given the detection of OCT4, HNK-1 and NGFR, cell induction for 6 days should be enough to produce NCCs from hiPSCs in our CDM.

To date, the detailed mechanisms of CEC development and the conversion of NCCs into the ocular anterior segment are still unclear. Hence, we selected 30 compounds, including those that affect signaling pathways related to corneal development and stem cell differentiation, to build this protocol. Neural crest migration has been reported to start with the process of epithelial-mesenchymal transition (EMT) with TGFβ signaling or WNT signaling (Saika et al., 2001; Zacharias and Gage, 2010). In this study, A769662 and AT13148 were screened and showed the most efficient CEC induction. A769662, a potent and reversible activator of AMPK, affects CEC differentiation by activating AMPK (Cool et al., 2006). AMPK is a conserved serine/threonine kinase that functions as an intracellular energy sensor to maintain energy balance and metabolism and enhance the migration and differentiation of endothelial cells (Carling, 2004; Li et al., 2008; Liu et al., 2011; Reihill et al., 2011). AT13148 is an orally active and ATP-competitive inhibitor of multiple AGC kinases, which play important roles in cell proliferation and survival (Vasudevan et al., 2009; Pearce et al., 2010; Manning and Toker, 2017). Apparently, the A769662 and AT13148 have opposite roles in regulation the AMPK and PKA kinases. But AT13148 show the activation of PKA instead of the inhibition in this study which is also reported by Okuzumi *et al.* The potential mechanism that AT13148 induced Akt hyperphosphorylation (Thr308) could be re-localization of the AKT to the cell membrane (Okuzumi et al., 2009). AT13148 was hijacked to activate the PKA/Akt signaling in the NCCs of this study. Therefore, we hypothesize that AT13148 enhances the phosphorylation of Akt to promote FoxO1 in order to promote the expression of PITX2. Moreover, A769662 participates in endothelial cell differentiation by interacting with AT13148 to affect PAX6 and FOXC1.

For the final differentiation and maturation of hiPSC-CECs in our protocol, EGF and CHIR99021 were added to the conditioned medium to promote the maturation of hiPSC-CECs. The CECs displayed a regular hexagonal morphology and tight junctions. EGF is mitogenic and stimulates CEC migration and wound closure (Joyce et al., 1989) (Huo et al., 2015). CHIR99021, an inhibitor of GSK-3, activates the WNT signaling pathway, which plays an integral role in the differentiation and development of cells and tissues (Cohen and Goedert, 2004). According to a previous study, hiPSC-CECs should express CEC markers, including ZO1, AQP1, and Na^+^/K^+^-ATPase (McCabe et al., 2015; Zhao and Afshari, 2016). Combination treatment with EGF and CHIR99021 can maintain the stable status of hiPSC-CECs. Thus, this CDM-based protocol should be efficient and applicable to produce specific CECs from patients in the clinic.

## Conclusion

We induced hiPSCs to differentiate into human corneal endothelial-like cells with CDM. A small-molecule library was used to screen the best small molecules (AT13148 and A769662) to promote the conversion of human NCCs into the ocular anterior segment and CECs.

## Data Availability

The original contributions presented in the study are included in the article/Supplementary Material, further inquiries can be directed to the corresponding authors.
