# Supplementary material for: Small-Molecule Induction Promotes Corneal Endothelial Cell Differentiation From Human iPS Cells
Source: Front Bioeng Biotechnol. 2021 Dec 15;9:788987. doi: 10.3389/fbioe.2021.788987 (PMC8714889; doi:10.3389/fbioe.2021.788987)
Supplement: Supplementary file 1 [file DataSheet1.docx]

Supplementary Material

# Supplementary Tables

**Supplementary Table1: Primers used in RT-PCR experiments**

| Genes | **Forward (5’-3’)** | **Reverse (5’-3’)** |
| --- | --- | --- |
| OCT4 | CCCCAGGGCCCCATTTTGGTACC | ACCTCAGTTTGAATGCATGGGAGAGC |
| SOX9 | GGCCAACCTTGGCTAAAT | CACACGATTCTCCATCATCC |
| SOX10 | CAGTCCTCCTCCACTACAA | GAGGCTCTGTGAATTGTCTC |
| NTRK3 | TGGAAGGGCAGGATTCA | CGCCAGTTCTCTATGTGTATG |
| NGFR | TAGGACTCTGAGGCTCTTTC | CAGAGGCTTTCCACAACTC |
| ZO1 | TGGACGGGCACAACTTCATC | GGGCAGGTTCTTGGCACTCT |
| COL8A1 | GCAAAGAGTATCCACACCTAC | CCCTCGTAAACTGGCTAATG |
| AQP1 | CATTTAGAGGGTGAAGGAGAAA | GAGGGAGTAGAGAACTGAAGA |

**Supplementary Table2: Antibodies used in immunostaining and WB**

| **Antibodies** |  | **Source** | **Dilution** |
| --- | --- | --- | --- |

| **Immunostaining** |  |  |
| --- | --- | --- |
| PAX6 | Covance, PRB-278P | 200 |
| SOX2 | Millipore, AB5603 | 500 |
| OCT4 | Millipore, AB3209 | 500 |
| TRA-1-60 | Abcam, ab16288 | 200 |
| NANOG | Proteintech, 14295-1-AP | 200 |
| SSEA4 | Abcam, ab16287 | 200 |
| NGFR | CST, 8238 | 200 |
| B3GAT1 | Proteintech, 19401-1-AP | 200 |
| VIMENTIN | Millipore, AB1620 | 200 |
| AQP1 | Proteintech, 20333-1-AP | 200 |
| ATP1A1 | Proteintech, 14418-1-AP | 200 |
| ZO-1 | Invitrogen, 61-7300 | 500 |
| Alexa conjugated secondary antibodies | Thermo Fisher Scientific | 500 |
| **Western blot** |  |  |
| P-Akt(Thr308) | CST, #13038 | 1000 |
| Akt | CST, #9272 | 1000 |
| P-AMPK𝛼(Thr172) | CST, #2535 | 1000 |
| AMPK𝛼 | CST, #2532 | 1000 |
| PITX2 | Proteintech, 67202-1-lg | 1000 |
| FOXC1 | Proteintech, 55365-1-AP | 1000 |
| P-FoxO1 (Thr24)/FoxO3a (Thr32) | CST, #9464 | 1000 |
| P-FoxO1 (Ser256) | CST, #9461 | 1000 |
| P-FoxO1 (Thr24)/FoxO3a (Thr32)/FoxO4 (Thr28) | CST, #2599 | 1000 |
| FoxO1 | CST, #2880 | 1000 |
| P-FoxO3a (Ser253) | CST, #13129 | 1000 |
| P-FoxO3a (Ser318/321) | CST, #9465 | 1000 |
| FoxO3a | CST, #12829 | 1000 |
| FoxO4 | CST, #9472 | 1000 |
| HRP-conjugated β-Actin Antibody | Proteintech, HRP-60008 | 2000 |
